# Patients' Perception of Morocco's Medicine Pricing Reform and Determinants of Their Access to Health Care and Medicine

**DOI:** 10.1155/2021/6969333

**Published:** 2021-10-04

**Authors:** Amine Cheikh, Meryem Moutahir, Ismail Bennani, Houda Attjioui, Wadie Zerhouni, Mustapha Bouatia

**Affiliations:** ^1^Department of Pharmacy, Cheikh Zaid Hospital, Abulcasis University, Rabat, Morocco; ^2^Laboratory of Analytical Chemistry, Faculty of Medicine and Pharmacy of Rabat, Mohammed V University, Rabat, Morocco; ^3^National School of Public Health, Rabat, Morocco; ^4^Pediatrics Hospital, Ibn Sina University Hospital, Rabat, Morocco

## Abstract

**Background:**

In 2014, the Ministry of Health of Morocco implemented a reform of medicine pricing that leads to lower prices. This reform has brought about a new method of pricing medicines and a reduction in the prices of more than 1,400 of the 5,000 medicines on the market. The objective of this study was to survey patients' perceptions of the impact of the reform on medicine prices and affordability of health care, including medicine.

**Methods:**

Between September 2017 and September 2018, 360 patients that visited a community pharmacy in four selected areas of different socioeconomic levels were interviewed based on a questionnaire. Findings were studied through univariate and multivariate analyses.

**Results:**

Three hundred patients (83%) were included given their completed questionnaire. The majority (89%) of respondents considered medicine prices as a potential barrier to access to health care. Lower medicine prices following the reform were not perceived to have actually impacted respondents' spending on health care. In some cases, care was delayed, in particular by lower-income respondents and people without insurance and health coverage.

**Conclusion:**

The majority of patients participating in the study did not perceive the decrease in medicine prices as sufficient. In addition, the study findings pointed to the relevance of further determinants of access to medicines, such as health insurance coverage. Patients think that the generalized third-party payment mode, which does not oblige patients to spend out of their pockets to have their treatment but rather their health insurance funds that will pay for them, provides optimal access to medicines.

## 1. Introduction

Medicine spending represents about 20% to 60% of health expenditures in most developing and transition countries [1], against an average of 18% in the Organization for Economic Cooperation and Development (OCDE) countries where medicines' expenditures represent between 6.4% and 35.4% [2]. Medicine spending also has a large variation across countries in the same income group, expressing the importance of both the way medicines are priced and the capacity of governments to implement specific cost-sharing policies to influence out-of-pocket spending on medicines [3]. In middle-income countries as well as in low-income or high-income countries, medicines absorb a greater share of primary health care spending [4].

Out of pocket (OOP) is the main source of funding for almost 90% of the population in developing countries [5]. OOP payments can constitute a major barrier for affordable access to health care, including essential medicines [6–11].

The main objective of medicine price regulation is to contain (public and private) health expenditure to ensure affordable medicine prices. In Morocco, medicine price policy is one of the pillars of the national pharmaceutical policy. Medicine price regulation was established in Morocco in 1969 with different provisions for locally manufactured products and imported medicines.

In 2014, the Ministry of Health implemented a reform on medicine pricing and changed provisions on how to determine medicine prices in Morocco. This was done in reaction to study reports and surveys conducted during the first decade of the 21^st^ century. The first one was a WHO survey on medicine prices in collaboration with the Ministry of Health in 2004, followed by a report of the Parliamentary Information Mission on the prices of medicines, presented by the Committee of Finance and Economic Development of the parliament in 2009. In 2010, the Competition Council performed a study on the competitiveness of the pharmaceutical industry. These studies concluded that the prices of medicines are relatively high in comparison with countries of similar income.

The main changes introduced by the new decree are summarized below:

- Introducing external price referencing based on seven reference countries (Spain, Portugal, France, Belgium, Turkey, Saudi Arabia, and the country of origin of the medicine) for new medicines and a generic's price link policy to branded medicines for generics (the price must not exceed a maximum reference price based on the price of the branded medicine by applying a discount on this medicine's price) [12]
- Harmonizing rules applicable to both local manufactured and imported medicines
- Changing the wholesale and pharmacy margins from linear margins to a regressive margin scheme according to the manufacturer prices excluding taxes (the higher the price of the medicine, the lower the distribution margin)

Following the implementation of this pricing decree in 2014 and until the end of 2019, the prices of 2,572 medicines were revised and subsequently decreased, followed by price reductions for certain medicines at some later points in time. This price revision addresses all price components (e.g., ex-factory price, wholesale price, pharmacy, retail price, net, and gross) and thus has an impact on all actors in the pharmaceutical chain, namely, pharmaceutical companies, wholesalers, dispensing pharmacies, and ultimately the patients.

This study surveyed the perceptions of patients whether or not and how they experience the drop of medicine prices and if this strategic government reform contributed to improving households' access to medicine.

## 2. Methods

A questionnaire survey, in French and Arabic languages, with face-to-face interviews was conducted among 360 patients visiting community pharmacies in the private sector between September 2017 and September 2018. The questionnaire was established and validated by a team of pharmacists and a health economist. Pharmacists in cooperating selected pharmacies were asked to interview patients who visited the pharmacies based on this questionnaire. No data to identify patients was collected, and the questionnaire was managed anonymously throughout this study.

Patients included in the study were those who presented a prescription containing at least one medicine that had undergone a price reduction or those who came to the pharmacy with the intention to purchase at least one medicine that had undergone a price decrease as part of the price reform.

A cluster sampling of community pharmacies was carried out in such a way that it covers the various socioeconomic areas of the city of Rabat, in neighborhoods of high socioeconomic level, middle socioeconomic level, and low socioeconomic level at the rate of 2 pharmacies per locality. Subsequently, the pharmacies most frequented by patients were selected in the cases that they agreed to participate in the study.

Questionnaires completed by pharmacists following their interviews with patients who agreed to participate in the study were entered into SPSS 13.0 software to perform the necessary statistical analyses.

The questionnaire included a total of 28 questions that related to the socioeconomic status of the patient, their health and their health service utilization, the financial burden of health care and medicine, the perception of changes in medicine prices, and personal strategies to deal with possible high costs (Appendix).

The comparison was made using the chi^2^ test for qualitative variables. Univariate and multivariate analysis was performed using binary logistic regression to understand the variables that influence the accessibility of medicines after the price drop. The variables that were studied in the univariate and multivariate analysis are age, sex, health insurance coverage, employment, family situation, origin, and monthly income.

## 3. Results

Of the 360 patients who were the target of the study, the sample included 300 patients who agreed to participate and whose data are completely and correctly filled. Sixty questionnaires were excluded because of the incompleteness of the participant's responses or withdrawal. All characteristics and answers of the participating population are summarized in Table 1.

**Figure 1:**
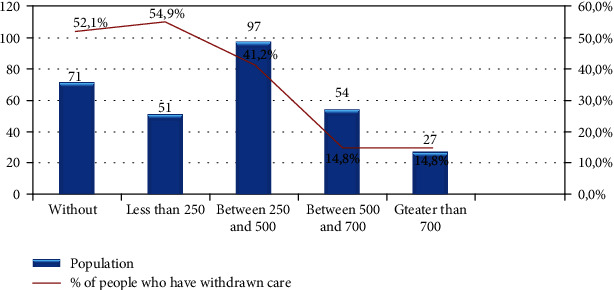
Share of interviewed people who postponed their health care by income.

**Figure 2:**
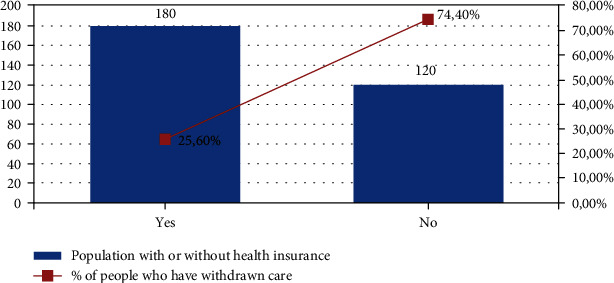
Comparison between the percentage of the delay in health care in people who have or no medical coverage.

**Figure 3:**
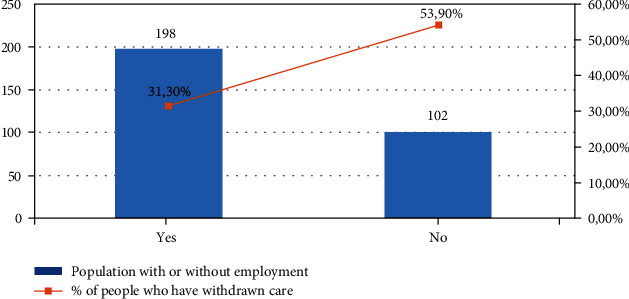
Comparison between postponing care based on having a job.

39% of respondents were forced to postpone or even give up their care in the last 12 months mainly due to lack of financial means. Postponement of care was, in particular, a problem for those with low income, those without social health insurance coverage, and those without employment (Figures 1–3).

The response of participants to a change in price differed significantly depending on whether or not the patients were covered by medical insurance coverage, they have or not, the employment, their origin, and their income. The decrease in prices was rather perceived by people without health insurance (66.3%), unemployed people, and people in the urban environment.

The multivariate analysis of the factors associated with a perceived improvement in the accessibility of medicines after the decline in their prices showed that health insurance coverage affiliation is the principal factor that was associated to feel that a decline in medicine prices may have improved accessibility to medicines (*P* = 0.002) (Table 2).

## 4. Discussion

This study assessed patients' perception of the decline of medicine prices and provided an idea of the impact of this strategic government reform on household accessibility of medicines. To our knowledge, this is the first study conducted on patients' perception since the fall in prices undertaken by the MOH in 2014. Some other studies have been conducted, but not published, to assess the impact of this reform on other stakeholders of the pharmaceutical sector in Morocco: e.g., national and international pharmaceutical industry, pharmaceutical wholesalers and distributors, pharmacists, and mandatory health insurance funds.

The results of this study showed that even though the majority of the study population is in urban areas, 1/3 did not have a job and was struggling to find ways and means to cover their health care costs. However, the proportion of the population that had medical coverage was 60% of our sample; the majority is mainly affiliated with the mandatory health insurance (MHI) regime managed by two funds: the National Fund of Social Welfare Organizations (CNOPS) and the National Social Security Fund (CNSS) [13–16]. This percentage reflects the percentage of the Moroccan population currently covered by a basic medical coverage scheme, which is estimated at between 60% and 65%. This population is covered either by the MHI, by the medical assistance scheme, or by private insurance. Thus, 40% do not have medical coverage and fully pay out-of-pocket expenses related to their health care. This proportion should draw our attention to this population still not covered by a basic medical coverage (BMC) even after 15 years of the establishment of social health insurance in Morocco. Income is undoubtedly a fundamental element in accessibility to care; the higher the income, the better the accessibility, and vice versa. That is, what we found that the more the people have low incomes, the more they tend to postpone health care services and endure the disease.

A third of our interviewed population considered the cost of care excessive; this high cost pushed 17% of the population of the study to spend nothing on their health because of the lack of means in this population. In addition, the cost of care has pushed 39% of the population to postpone their care because of their low income, lack of medical coverage, or because of their unemployment. This postponement of care could be at the origin of worsening of the patients' state of health and additional costs of their care [17–19].

Regarding the decrease in the price of medicines following the reform and dissemination activities of the Ministry of Health on this policy change, the findings of this survey suggest that the Ministry has communicated well on this subject since 67% of the population studied have taken note of this decline in the prices of medicines.

Medicine prices were decreased in 2014 because studies had shown that they were high, even in comparison with other countries. These findings were confirmed by the perception of the interviewed people because 3 out of 4 considered medicine prices before the reform as excessive. In the same way, the majority of respondents (89%) considered that the price of medicines could be a barrier to patients' access to care, which is why this policy was introduced and implemented despite all the obstacles encountered by the MOH.

However, the decrease in prices was not considered sufficient because only 1/3 of our population felt that there was a decline in drug prices; the majority felt that they did not feel the impact of this decline in prices on their expenses. This result leads us to believe that the policy undertaken by the MOH has not had the expected results. In addition, only half of the population felt that they had better access to medicines after the price of these drugs dropped.

Furthermore, those without any health insurance coverage in particular expressed the perception of insufficient and even nonexisting decreases in medicine prices, as they have to pay fully out of pocket for health care services, including medicines. On the other hand, those that have social health insurance benefit from lower prices since their copayment (which is linked to the medicine price) are lower.

The univariate and multivariate analysis of the parameters that could improve the accessibility of the study population to medicines, after the decline in their prices, has shown that a single parameter that could improve this accessibility was the affiliation to medical coverage. Thus, the results confirmed the existence of social health insurance coverage as a major determinant to ensure access to health care, including medicine. In the same way and concerning affordability, it has been announced that for households, high out-of-pocket payments can have clinical repercussions (in particular, those in need forego or interrupt their treatment), economic repercussions (high out-of-pocket expenditures for medicines reduce household spending on other necessary items), and societal repercussions (e.g., community divisions stemming from inequitable medicine access due to cost) [20].

In addition, the findings strongly suggest that the design of the coverage scheme is key. Study participants even attributed higher importance to the third-party payment than to reduced medicine prices; several solidarity-based health care systems in high-income countries organized social health insurance on such a system [21].

In Morocco, the health insurance scheme (mandatory health insurance) has also opted for this method of payment via health insurance funds. An agreement has been signed in 2012 between the pharmacists and the health insurance funds. This agreement has established a limited list of medicines covered by this agreement (just 120 medicines among 4,200 reimbursable medicines). The list includes expensive drugs or drugs leading to high cost of treatment. Other medicines should be integrated into this list to allow better accessibility of patients to drugs.

Furthermore, we cannot talk about accessibility to drugs without talking about the importance of generic medicines. Generics help ensure access to medicines due to their low prices. Public sector availability of generic medicines is less than 60% across WHO regions, ranging from 32% in the Eastern Mediterranean Region to 58% in the European Region. However, the availability is still less than 60% in the Western Pacific, South-East Asia, and Africa Regions [22]. In the US, the richest country in the world, generic drugs typically capture 80-90% of sales of a given drug in the year after entry, thanks in part to generic substitution and other policies used by payers to promote the use of generic drugs [23]. Retail price decreases resulting from the entry of generic drugs are based on the number of competitors and can reach up to 90% of the brand price before launch [24]. This is unfortunately not the case in many developing and middle-income countries where the generic drug represents only 29.4% to 54.4% [25]. In Morocco, the part of generic is about 80% in the public sector and 39% in the private sector. Several initiatives have been undertaken but much remains to be done to improve the introduction of generics into medical prescription and patient consumption.

Our work has some limitations:

- First sample of the population was limited due to the difficulty of retaining patients for thirty minutes to explain the questionnaire and fill it later
- Secondly, we could not access pharmacies in rural areas or in remote localities to measure the perception of this population on the drop in drug prices and to assess whether this population had better access to health products after this price drop
- Third, addressing only patients who visit pharmacies to buy drugs to answer questionnaires will probably exclude those who cannot buy them and therefore do not visit private pharmacies
- Another point is that concerning the report of care of the patients. It should have been more interesting also to assess whether patients have completely canceled treatment or simply postponed

## 5. Conclusion

The principal conclusion of our article is the decrease in prices was not felt and not perceived sufficiently by population. However, the generalization of medical insurance coverage to the entire population is undoubtedly the key factor to improve access to medicines and health services, especially in resource-limited countries.

## Figures and Tables

**(a):** 

Quantitative variable	Median, interquartile
Age	45 [31, 59]
Pharmaceutical expenditure (in euros)	30 [20, 60]
Blood and urinary analyzes expenditures (in euros)	15 [0, 50]
Doctor's visit expenditures (in euros)	25 [0, 30]
Total care expenditures (2016) (in euros)	400 [200, 700]

**(b):** 

Qualitative variable	Number (percentage)
Sex	
M	127 (42.3%)
F	173 (57.7%)
Education level	
Illiterate	32 (10.7%)
Primary	72 (24%)
Secondary	120 (40%)
University	76 (25.3%)
Health insurance coverage	
Yes	180 (60%)
No	120 (40%)
Family status	
Married	190 (63.3%)
Single	88 (29.3%)
Other	22 (7.3%)
Employment	
Yes	198 (66%)
No	102 (34%)
Origin	
Urban	242 (80.7%)
Rural	58 (19.3%)
Monthly income	
Without income	71 (23.7%)
Less than 250 €	51 (17%)
Between 250 € and 500 €	97 (32.3%)
Between 500 € and 700 €	54 (18%)
Greater than 700 €	27 (9%)
Type of provider	
General practitioner	119 (39.7%)
Specialist	161 (53.7%)
Never	20 (6.7)
Frequency of medical visit	
Regularly when I am sick	156 (52%)
For chronic disease monitoring	112 (37.3%)
When I am sick and I have money	32 (10.7%)
Chronic diseases	
Yes	126 (42%)
No	174 (58%)
100% coverage of healthcare expenses	
Yes	12 (4.1%)
No	278 (92.7%)
Estimated monthly amount of health expenses	
Yes	178 (59.3%)
No	122 (40.7%)
Delaying care	
Yes	117 (39%)
No	183 (61%)
Share of health expenditures in family expenditures	
Nothing	50 (16.7%)
Excessive	99 (33%)
According to needs and means	46 (15.3%)
Moderate	70 (23.3%)
Strictly necessary	34 (11.3%)
Informed on drop of drug prices	
Yes	201 (67%)
No	99 (33%)
Costs of care before the prices drop	
Expensive	166 (55.3%)
Moderate	75 (25%)
Insignificant	59 (19.7%)
Costs of care after the prices drop	
Decrease in expenses	52 (17.3%)
Moderate	109 (36.3%)
No change	138 (46%)
Realizing that medicine prices are lower than in the past	
Yes	86 (28.7%)
Moderately	79 (26.3%)
No	135 (45%)
Abnormally high price	
Yes	215 (71.7%)
No	28 (9.3%)
Some medicines	57 (19%)
The price of the medicine is a barrier to access medicines and health care services	
Yes	266 (88.7%)
No	34 (11.3%)
Better access to medicines after drop of prices	
Yes	147 (49%)
No	152 (50.7%)
Significantly lower	
Yes	130 (43.3%)
No	170 (56.7%)
Lower prices did not improve access to health care services for patients	
Yes	126 (42%)
No	173 (57.7%)
Do you think that the impact of the “tiers payant” on medicine accessibility is more important than decrease medicine prices NB: “tiers payant” (i.e., direct payment of the health care providers by the payer instead of the reimbursement)	
Yes	271 (90.3%)
No	29 (9.7%)

**Table 2:** Univariate and multivariate analysis of the factors associated with an improvement in the accessibility to medicines after the decline in their prices.

Independent variables	Univariate analysis			Multivariate analysis		
	*β*	95% IC	*P*	*β*	95% IC	*P*
Age	0.004	0.001–0.007	0.020	0.003	0.000–0.006	0.059
Sex						
Male	0					
Female	-0.047	-0.162–0.067	0.421			
Health insurance						
Yes	0					
No	0.209	0.096–0.322	<0.001	0.187	0.066–0.307	0.002
Employment						
Yes	0					
No	-0.047	-0.166–0.073	0.422			
Family situation						
Married	0					
Single	-0.088	-0.309–0.132	0.433			
Other	-0.091	-0.324–0.142	0.445			
Origin						
Urban	0					
Rural	-0.161	-0.303-0.019	0.027	-0.064	-0.213–0.085	0.400
Monthly income						
Without income	0					
Less than 250	0.011	-0.208–0.231	0.918	-0.077	-0.301–0.147	0.500
Between 250 and 500	0.166	-0.066–0.397	0.161	0.053	-0.183–0.290	0.659
Between 500 and 700	0.003	-0.208–0.214	0.977	-0.051	-0.261–0.159	0.633
Greater than 700	-0.029	-0.258–0.201	0.807	-0.059	-0.284–0.165	0.604

## Data Availability

Data are available from the authors and will be provided on request.

## Appendix

1. Sex
2. Age
3. Education level (primary, secondary, university)
4. Medical coverage
5. Family situation (married, single, other)
6. Employment
7. Geographic origin (urban, rural)
8. Monthly income (without income, less than 250 $, between 250 $ and 500 $, between 500 $ and 700 $, greater than 700 $)
9. Ambulatory care: Do you consult a doctor? (general practitioner, specialist, never)
10. Doctor's visit (when I am sick regularly, for monitoring a chronic disease, when I am sick and I have the financial means)
11. Chronic diseases: Do you suffer from any chronic disease?
12. Do you benefit from 100% reimbursement of expenses incurred?
13. Can you estimate the monthly amount of your health expenditures?
14. If yes, what is this amount for (approximately) (drug, biological analysis, consultations, radiology)
15. Access to care: Have you been delaying any health care in the last 12 months?
16. For what reason (s)? (Financial, nonavailability of the service, quality of care)
17. Total health care cost in 2016? (Global package; medicines not reimbursed; care not reimbursed; medical device, prosthesis, etc.; hospitalization; hospital package; overruns; miscellaneous expenses; medical transport; other (specify))
18. Share of your health expenditures compared to your income/budget (nothing, I cannot afford health care for myself, excessive, big part of my budget, I spend according to my needs and my capacities, moderate, the bare necessities)
19. The Ministry of Health has made a further decrease of some drugs marketed in Morocco. Are you aware of this decrease?
20. Perception of costs of care before the price drop of medicines (expensive, average, insignificant)
21. Costs of care after the price drop of drugs (decrease in expenses, more or less, no change)
22. Did you feel this price drop on your expenses?
23. Do you think that the price of medicines in Morocco is excessive?
24. Is the price of medicines a real barrier to access to health care?
25. After this drop in medicine prices, is access to your treatment better? Any improvement?
26. Was the drop significant enough?
27. Do you think that it does not bring any improvement to patients?
28. Do you think that the “tiers payant” system (the patient is not obliged to pay the cost of his medications to the pharmacist. It is the health insurance fund that will reimburse the pharmacist after dispensing medication to the patient) could improve your accessibility to medicines?
